# Glioblastoma vaccines: past, present, and opportunities

**DOI:** 10.1016/j.ebiom.2023.104963

**Published:** 2024-01-05

**Authors:** Zujian Xiong, Itay Raphael, Michael Olin, Hideho Okada, Xuejun Li, Gary Kohanbash

**Affiliations:** a Department of Neurological Surgery, University of Pittsburgh School of Medicine, Pittsburgh, PA 15201, USA; b Xiangya School of Medicine, Central South University, Changsha, Hunan 410008, PR China; c Department of Pediatrics, Masonic Cancer Center, University of Minnesota, Minneapolis, MN 55455, USA; d Department of Neurological Surgery, University of California, San Francisco, CA 94143, USA; e Department of Neurosurgery, Xiangya Hospital, Central South University, Changsha, Hunan 410008, PR China; f Hunan International Scientific and Technological Cooperation Base of Brain Tumor Research, Xiangya Hospital, Central South University, Changsha, Hunan 410008 PR China; g Department of Immunology, University of Pittsburgh, Pittsburgh, PA 15213, USA

**Keywords:** Glioblastoma, Vaccine platform, Tumour antigen, Vaccine efficacy, Vaccine perspective

## Abstract

Glioblastoma (GBM) is one of the most lethal central nervous systems (CNS) tumours in adults. As supplements to standard of care (SOC), various immunotherapies improve the therapeutic effect in other cancers. Among them, tumour vaccines can serve as complementary monotherapy or boost the clinical efficacy with other immunotherapies, such as immune checkpoint blockade (ICB) and chimeric antigen receptor T cells (CAR-T) therapy. Previous studies in GBM therapeutic vaccines have suggested that few neoantigens could be targeted in GBM due to low mutation burden, and single-peptide therapeutic vaccination had limited efficacy in tumour control as monotherapy. Combining diverse antigens, including neoantigens, tumour-associated antigens (TAAs), and pathogen-derived antigens, and optimizing vaccine design or vaccination strategy may help with clinical efficacy improvement. In this review, we discussed current GBM therapeutic vaccine platforms, evaluated and potential antigenic targets, current challenges, and perspective opportunities for efficacy improvement.

## Introduction

Glioblastoma (GBM) is one of the most aggressive malignancies of the central nervous system (CNS) tumours. Standard of care (SOC) therapy for GBM includes maximum safe surgical resection followed by adjuvant radiotherapy, temozolomide (TMZ) chemotherapy, and tumour-treating fields (TTF). These treatments (with the exception of TTF) have remained unchanged for almost 20 years without vital progress and median overall survival (OS) from diagnosis of roughly 15 months.^1^, ^2^, ^3^ Most GBM patients experience tumour recurrence.^4^, ^5^, ^6^ The high recurrence rate can be attributed to unresectable sites of quiescent tumour seeding cells distantly located from the initial tumour at the early stage of tumorigenesis^7^ or tumour-normal brain boundary-infiltrated tumour cells.^8^ The most recent World Health Organization (WHO) criteria classifies adult malignant gliomas as astrocytoma, IDH-mutant (grades II, III, or IV), Oligodendroglioma, IDH-mutant, 1p/19q-codeleted (grades II, III), and Glioblastoma, IDH-wildtype (grade IV). Important to note, non-GBM gliomas can also recur as more malignant and lethal higher-grade gliomas.^9^ Insufficient treatment allows these residual tumour cells to form a virtual nuclear and cytoplasmic continuum with adjacent normal cells,^10^ whereby they introduce genetic elements and abnormal proteins to change normal cell phenotype by directly forming gap junctions^11^ or releasing tumour-derived extracellular vesicles,^12^ as well as rescue damaged tumour cells. Overall, new supplementary therapies are necessary to further decrease the unobservable tumour load and attenuate tumour progression. Provoking host immunity using immunotherapy has long been thought promising to achieve a better clinical outcome in GBM management. Integrating immunotherapy into SOC of GBM is a feasible next step to improve treatment efficacy for GBM patients.^13^

There are several modes of immunotherapy. Prior work has initiated such type of distinction for the domain of neuro-oncology^14^ including the concepts of 1) restorative immunotherapy (e.g., cytokine stimulations), 2) adoptive cell transfer immunotherapy (e.g. chimeric antigen receptor T cells (CAR-T) and T cell receptor-engineered T cells (TCR-T)), 3) passive immunotherapy (antibodies), 4) active specific immunotherapy (vaccines), 5) modulatory immunotherapy (amongst others the checkpoint inhibitors), and 6) immunogenic cell death (ICD) therapy (e.g. tumour-treating fields, modulated electrohyperthermia, oncolytic virus therapy).^15^^,^^16^ Other concepts related to immunotherapy include those not associated with a concrete patient phenotype (such as ICD therapy), those personalized in a manner that an antigen present on that patient’s tumour is the target, or individualized immunotherapies that account for the presence of a target and the patient’s immune identity such as autologous DC vaccines or adoptive cell transfer of tumour-infiltrating leukocyte therapy (TIL therapy). While these definitions have been evolving, the basis remains the same.

According to antigen dependency, immunotherapies can be separated as antigen-independent therapies, such as indoleamine 2,3-dioxygenase 1 (IDO) inhibitor, oncolytic viral therapy, and immune checkpoint blockade (ICB), and antigen-specific approaches, including CAR-T, TCR-T, and vaccines.^17^ The primary distinction between these antigen dependencies is not whether tumour antigens are required but rather whether a known antigen or set of antigens is being targeted. Antigen-independent therapies often aim to block the immunosuppressive mechanism within the tumour microenvironment (TME) to assist T cells to escape exhaustion, activate antitumor immunity, or facilitate tumour antigen release. To date, no results from clinical trials of GBM IDO inhibitor treatment have been reported (NCT02052648, NCT04047706, and NCT02502708). Despite dramatic successes against a variety of solid tumours,^18^, ^19^, ^20^ several phase III clinical trials on GBM ICB therapies have reported no improvement in OS.^21^, ^22^, ^23^ The efficacy of neoadjuvant ICB immunotherapy in patients with GBM is controversial. Some researchers have reported that neoadjuvant ICB immunotherapy promotes OS in patients with recurrent GBM^24^; however, no significant change in cytotoxic T-cell function or immune checkpoint expression was reported in others,^25^^,^^26^ contributing to an unsatisfactory survival benefit in GBM patients. Overall, the limited efficacy of ICB as a monotherapy supplement to SOC has been attributed to the ‘cold’ (immunologically inert) TME,^27^ GBM-induced tumour-supportive phenotypic modifications,^10^ and low tumour mutation burden (TMB) of GBM,^28^^,^^29^ which has been recently reported to be derived from the abnormal epigenome.^30^ High mutational load is typically essential for successful ICB treatments in some types of cancers,^31^^,^^32^ however, a recent study reported that very low TMB in recurrent GBM patients is associated with longer survival after ICB treatment.^33^ Furthermore, oncolytic virus pre-treatment can effectively promote ICB treatment efficacy by increasing antigen exposure.^34^, ^35^, ^36^ Therefore, enrolment of antigen-specific immunotherapy in a patient treatment regimen can improve clinical outcomes.

In this review, we broadly discuss antigen-specific immunotherapies for brain tumours, with a focus on therapeutic vaccine types and platforms for GBM vaccination. We also outline current challenges, ongoing strategies, and perspectives for improving GBM immunotherapy in the clinic.

## Antigen-specific therapies

Recently, antigen-targeting therapy, using mRNA vaccines or TCR-T showed a favourable effect in promoting the regression of pancreatic cancers that acquired resistance to ICB.^37^^,^^38^ More than just targeting designed antigens, some patients treated with these antigen-targeting strategies exhibit antigen spreading to other non-targeting antigens.^39^^,^^40^ These studies provide a foundation for antigen-targeted therapies for checkpoint-refractory tumours.

Cancer vaccines aim to generate de novo tumour antigen-specific T cells through professional antigen-presenting cell (APC) presentation. Prophylactic (preventative) anti-viral vaccines to mediate cancer protection have been extremely successful.^41^, ^42^, ^43^ Furthermore, therapeutic vaccines against cervical intraepithelial neoplasia (CIN) associated with the high-risk human papillomavirus (HPV) subtype, HPV-16 and HPV-18, can significantly increase the rate of histopathological regression by around 20%^44^ and even induce a complete response.^45^ Although no vaccines have been reported to prevent non-viral cancer, some therapeutic vaccines show promise for regressing non-viral tumours and improving outcomes. Triple-negative breast cancer (TNBC) is the most aggressive form of breast cancer worldwide. Treating TNBC patients with the HER2 peptide AE37 vaccine demonstrated the clinical benefit potential of tumour vaccination.^46^ For GBM patients, the therapeutic benefit of single peptide vaccines performed poorly in phase III trials and remains controversial,^47^, ^48^, ^49^, ^50^ but some early-phase multi-peptide vaccines show favourable clinical efficacy as a single supplementary therapy for SOC.^51^, ^52^, ^53^, ^54^, ^55^ For cellular antigen-specific therapies, CAR-T cell clinical trials targeting GBM antigens (epidermal growth factor receptor variant III (EGFRvIII),^56^ human epidermal growth factor receptor 2 (HER2),^57^ and IL-13 receptor α2 (IL-13Rα2)^58^^,^^59^) demonstrated the feasibility, safety, and potential efficacy of this approach in GBM treatment.

In addition to promoting GBM regression as monotherapy, vaccination can augment the therapeutic activity of other immunotherapies, including both antigen-dependent and antigen-independent approaches.^60^ For example, amphiphile-ligand with conjugated tumour vaccine peptide^61^ is a novel antigen supplementary strategy to enhance CAR-T cell tumour regression efficacy in the pre-clinical murine EGFRvIII GBM model.^62^ It partitions into the membrane of resident APCs after trafficking from the blood to lymph nodes through injection, then robustly primes endogenous CD4+ and CD8+ T cell responses and further induces antigen spreading. The above evidence suggests that antigen-specific therapies, including vaccines, which are the focus of this review, may enhance antitumor immune responses and prolong survival after SOC. Next, we discuss vaccine platforms, antigen types, remaining challenges, and opportunities for therapeutic GBM vaccination.

## Vaccine platforms

**Fig. 1:**
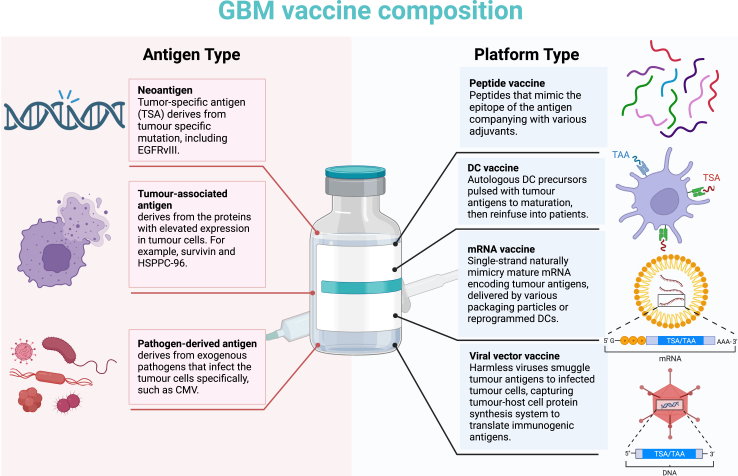
GBM vaccine composition. Left, the antigen type for the GBM vaccine. Right, the vaccine platform used in GBM vaccine delivery.

The general elements for vaccine design include an antigen, a danger signal, and a vehicle to transport the antigen. The combination of these three contributes make the potency and spectrum of the vaccine. Next, we discussed four vaccine platforms (Fig. 1), which incorporate these elements, that have been applied in clinical trials in vaccines for GBM: peptide vaccines, dendritic cell (DC) vaccines, mRNA vaccines, and viral vector vaccines.

### Peptide vaccines

Compared to cell-based vaccines, peptide vaccines can be produced faster and with fewer resources. These utilize tumour-specific, or tumour-highly-expressed peptide sequences synthesized chemically in vitro to immunize patients.^63^ There are two main types of therapeutic cancer peptide vaccines, minimal peptide epitopes (∼8–11 amino acids, AAs) or synthetic long peptides (SLPs, ∼25–30 AAs).^64^ The minimal peptide epitope could directly bind to the major histocompatibility complex (MHC) I-binding groove without an internalization process. However, it simultaneously raises the risk of binding to non-APC nucleated cells that express MHC I molecules but lack costimulatory molecules, which may lead to insufficient T-cell activation and even peptide tolerance.^65^ In contrast to the minimal peptide epitopes, SLPs require APC processing. Its long length allows APC to efficiently present both MHC I- (by cross-presentation) and MHC II-restricted epitopes.^66^ After in vivo injection, SLPs are engulfed and presented by APCs to autologous CD4+ and CD8+ T cells, and then these primed T cells circulate to the tumour and perform cytotoxicity. Owing to the lack of capability to activate the innate immune system, peptide vaccines need to be combined with additional immune adjuvants. Assist from adjuvants ensure sufficient costimulatory signals from APCs to elicit a robust T-cell response.^67^ The popular GBM vaccines are mainly peptide vaccines, such as rindopepimut (EGFRvIII) and SurVax (survivin).^68^ More details on current GBM peptide vaccines will be introduced in a later section.

### Dendritic cell (DC) vaccines

DCs are the most potent professional APCs to provoke adaptive immune responses. Before antigen uptake, DCs mainly existed during the immature stage. Antigen capture promotes DC maturation, with the secretion of various cytokines and upregulation of surface MHC and costimulatory molecules and cytokine receptors.^69^ DCs play pivotal roles in presenting tumour antigens in the lymph nodes to elicit T-cell priming and provoke distant antitumor response.^70^ Providing DCs with tumour antigens in (peptide vaccines) or ex vivo (DC vaccine) is of vital importance for inducing tumour-specific effector T cells. DC vaccines are generated ex vivo by culturing hematopoietic progenitor cells or monocytes with a combination of cytokines to induce mature DC differentiation. Then loading the selected tumour antigen with these DCs.^70^ In addition to priming CD4+ T cells by peptide-MHC II complex, these tumour antigen-loaded DCs also elicit effective CD8+ T-cell anti-tumour response through cross-priming, a process in which DC presents the exogenous peptide by MHC I molecules to prime CD8+ T cells.^71^ Compared to the whole protein directly coming from autologous tumour lysates, the synthetic peptides are more rapidly and efficiently processed by APCs, thus enhancing CD8+ T-cell activation.^66^ To guarantee an effective antitumor response, the existence of a DC co-stimulatory signal is an essential parameter. The lack of the necessary DC co-stimulation signals in the TME hampers DC maturation. Immature DC can present self-antigens to T cells, which leads to immune tolerance either through T cell deletion or immunosuppressive phenotype transformation.^72^ The additional inflammatory stimulus can help DC maturation and home to cervical lymph nodes^73^ and T cell immunostimulatory phenotype shift,^74^ improving vaccination efficacy. Compared to the peptide vaccine, the DC vaccine ensures DC maturation and high antigen-presenting efficiency with sufficient exogenous costimulatory signals, whereas DC maturation may be impeded by insufficient costimulatory signals in patients with a weak primary immune status when vaccinated with peptides. Recently, a phase III trial on DC vaccine addition to SOC, DCVax-L,^75^ revealed a significant extension of OS in both primary and recurrent GBM patients which was well-tolerated. Meanwhile, the phase I clinical trial of the DC vaccine targeting GBM stem-like cells (GSC) in both newly diagnosed and recurrent GBM patients^76^ reported encouraging results for stem-like cell targeting strategy, consistent with results of a previous DC vaccine phase II study targeting GSC marker, CD133.^77^ Beyond the standard mature DCs, an early-phase clinical trial revealed that α-type 1 polarized DC (αDC1) is another feasible DC type that can induce prolonged type-1 T-cell response more efficiently in GBM patients.^78^ Different from incubating selected tumour antigens with autologous DCs ex vivo, a Sweden research group induced a type 1 conventional DC (cDC1) phenotype in healthy human somatic cells^79^ and various human tumour cell lines^80^ to improve tumour heterogeneous antigen presentation, including GBM cell lines with favourable reprogramming efficiency.^80^ The reprogrammed cDC1-like cells engulfed tumour antigen from the TME, upregulated both MHC I complexes and costimulatory molecules, and secreted proinflammatory cytokine, such as IL-12 and IFN-γ, reverting immunostimulatory response due to tumour MHC I degradation.^81^ These tumour-APCs boost self-immunogenicity and CTL-killing susceptibility by presenting more endogenous and exogenous tumour antigens, driving anti-tumour immunity in vitro and in vivo. This strategy solves the human leukocyte antigen (HLA) restriction problem in vaccine design.

### mRNA vaccines

mRNA vaccines transduce mRNA into cells, especially APCs, to present translated peptides. It possesses the advantages of high potency, versatility, ease of development, and low manufacturing costs. mRNA can encode peptides that may be difficult to synthesize. Additionally, mRNA can induce type I IFN secretion as an intrinsic adjuvant, recognized by TLR3, 7, 8, and cytosolic RNA receptors.^64^ To mimic an mRNA with a naturally mature structure, the mRNA vaccine was designed as a single strand with a 5′ cap and 3′ poly(A) tail. The open reading frame (ORF) of the antigen-coding region is equipped with start and stop codons, flanked by untranslated regions (UTRs).^82^^,^^83^ It is either delivered directly using various routes, such as cationic liposomes, mRNA-packaging nanotechnologies,^83^ and viruses,^84^ or indirectly by in vitro reprogramming cells.^82^^,^^83^ Once uptake through endocytosis, the escaped mRNA from ubiquitous RNase uses the host’s protein synthesis system to translate tumour antigen proteins instantly.^83^ These proteins are either further trimmed by immunoproteasomes and presented by MHC I molecules, or secreted outside the cells and taken up by APCs to be presented by MHC II molecules or cross-presented in MHC I molecules.^82^^,^^83^ The mRNA vaccine is only transiently active and completely degrades. In GBM patients, mRNA either induces the host immune system by RNA-pulsed DC or is delivered directly by nanoparticles.^85^ Pre-clinical^86^ and early phase clinical trials^86^ showed that the mRNA has the potency to induce a favourable anti-tumour response in GBM animal models or patients and effectively prolongs overall survival. Clinical trials of mRNA vaccine for GBM are ongoing (NCT04573140 and NCT04573140) and no results have yet been reported. Nevertheless, one phase I/II nonrandomized trial in stage IV renal cell cancer patients vaccinated with mRNA that codes the same tumour antigens for GBM patients, including MAGE-1, Survivin, and HER-2, reported favourable clinical benefits.^87^

### Viral vector vaccines

There are diverse recombinant viral vectors that have been used to deliver antigens clinically.^88^^,^^89^ Unlike oncolytic viruses lysing tumour cells, viral vectors are genetically modified to insert antigen-encoding sequences and lose their toxicity to suit clinically safe applications. These virus vectors can infect the target cells and translate antigen peptides by the host protein synthesis system. Due to their capability to deliver multiple antigens and induce a robust adaptive immune response mimicking a natural infection, no exogenous adjuvants are needed.^88^ In addition to inducing a potent immune response through delivered antigens, the recipient’s immune system is also augmented by the viral pathogen-associated molecular patterns (PAMPs). However, long-term usage of viral vectors can induce anti-viral humoral immunity.^34^ No clinical trial results have been reported; however, clinical trials on viral vector vaccines targeting human cytomegalovirus peptide in GBM are ongoing (NCT03382977).

## GBM antigen classification

Optimal antigen selection is key to the efficacy of tumour vaccination. An ideal tumour antigen should possess high immunogenicity and be expressed specifically by almost all tumour cells. During maturation, T cells have to pass the negative selection in the thymic medulla, which will delete the potential self-reactive T cell with high-affinity TCR for the self-antigen presented on DCs or thymic epithelial cells (TECs).^90^ Only non-self-antigens distinguished from peptides present in normal peripheral tissues can elicit an effective T cell response. Furthermore, an ideal tumour antigen should be expressed abundantly and stably during tumour progression, or tumour cells can escape from exogenous tumour antigen-induced immunosurveillance due to antigen loss. Not all tumour-derived peptides exhibit antigenicity. Impacted by MHC restriction, only peptides that are at fitted length and have matched anchor residues allow being presented by MHC molecules. A peptide with high affinity to MHC molecules correlates with stronger T-cell anti-tumour activity.^91^ Likewise, the peptides’ immunogenicity is also influenced by MHC restriction that TCR recognizes both antigenic peptide and MHC molecules, which have been discovered with more than 25,000 alleles.^92^ Thus, tumour antigen candidates should meet the MHC limitation, possess both antigenicity and immunogenicity, and have tumour specificity and stable abundance. Tumour antigens can be divided into endogenous and exogenous types. Endogenous antigens are antigens that originate from tumour cell intracellular proteins, including neoantigens (tumour-specific antigen, TSA) and tumour-associated antigen (TAA). Exogenous antigens have recently gained attention for use in tumour vaccine therapy. These antigens are derived from the tumour cells that preferentially infect pathogens and are presented by tumour MHC to activate the T cell response.^93^^,^^94^ Of note, GBM bulk tumour-derived antigen sources including tumour lysate vaccines, such as GBM6-AD^95^ and AV-GBM-1,^96^ or tumour-mRNA vaccines,^86^ putatively include these categories of antigens and still not recognized classes of antigens. In the following section, we discuss each tumour antigen type in GBM in more detail (Fig. 1).

### Neoantigen in GBM

Neoantigens come from somatic mutations whose clones only exist in the tumour cells. Due to GBM’s low TMB and high heterogeneity of GBM, only one neoantigen, EGFRvIII, has been identified and evaluated as an off-the-shelf neoantigen approach in clinical trials related to GBM. Rindopepimut is a peptide vaccine designed from the EGFRvIII deletion mutation that occurs in roughly 20–30% of all GBM.^48^^,^^97^ Although its phase II clinical trial reported a promising beneficial potency,^48^ the phase III randomized multicentre clinical trial on rindopepimut failed to show the clinical benefits of the EGFRvIII vaccine.^47^ Roughly 60% of patients after treatment lose the EGFRvIII expression at recurrence,^47^^,^^56^^,^^98^ potentially resulting from the cancer immunoediting effects,^99^ however this has not been definitively determined. Nevertheless, the EGFRvIII vaccine can be used as a complementary therapy to other immunotherapies. Recently, a study on EGFRvIII vaccine-boosted CAR T therapy reported an enhanced response despite the antigen loss in the murine EGFRvIII GBM model.^62^ The adjuvant EGFRvIII vaccine induced antigen spreading^39^ and promoted endogenous DC to engulf more tumour antigens to reinforce T-cell activation.

#### Future neoantigen detection

Current GBM neoantigens are mainly selected based on single nucleotide mutations, which have only one amino acid difference in the mutated peptide.^55^^,^^100^ Despite their high privacy and heterogeneity, GBM neoantigens from structural variation (SV), frameshifting, gene fusion, and cancer-associated chromosomal abnormalities^101^ are actively being explored.

### Tumour-associated antigens (TAAs) in GBM

TAAs include 1) developmental antigens, expressed during embryonic development and decrease or are silent after birth, 2) cancer-testis antigens (CTAs), elevated in tumour cells and germ cells (e.g. testis) relative to other tissues and contributing to meiosis, abnormal chromosome segregation, and aneuploidy,^102^ and 3) tumour overexpressed antigens, proteins expressed at a very high level in tumours compared with healthy organs. Increased CTA expression in GBM cells has been shown to promote CD8+ T cell activation and cytotoxicity,^103^ and immunized GBM patients with TAAs can extend overall survival,^104^ indicating TAAs’ potential in GBM vaccination. A recent clinical trial on newly diagnosed GBM patients^54^ revealed that both neoantigens and TAAs resulted in effective immunotherapies, especially for tumours with low TMB. TAA usage solves the antigen universality problem caused by preexisting antigenic heterogeneity, and these antigens can be shared among various patients. Several clinical trials reported that GBM TAAs, including MAGE-1, HER-2, gp100, AIM-2, TRP-2, EphA2,^105^ survivin^50^, IL13Rα2,^58^ and heat-shock peptide protein complex-96 (HSPPC-96),^106^^,^^107^ possess favourable anti-tumour potency^51^, ^52^, ^53^ (Table 1). In contrast to GBM neoantigen vaccines, GBM TAA vaccines tend to be in the form of combinations of peptides, such as SL-701,^51^ ICT-107,^52^ and IMA950,^53^ for clinical trial usage to compensate for their lower immunogenicity compared to neoantigens.^108^ Telomerase (TERT) is a major oncogene responsible for primary brain tumours, and its promoter mutation is found in approximately 80% of primary GBM cases, making TERT-derived peptides an ideal target for GBM neoantigen vaccines. Phase II clinical trials immunizing GBM patients with TERT mRNA-transfected DC (NCT03548571) or TERT-derived helper peptides (NCT04280848) as supplements to SOC are ongoing. GSCs play a pivotal role in tumour initiation, progression, and therapy resistance.^109^^,^^110^ It shows similar heterogeneity^111^ to different single-cell subtypes.^112^ GSCs highly express TAAs, mainly CTAs^113^ and MHC molecules,^114^^,^^115^ which strongly activate CD4+ and CD8+ T cells. TAAs, such as CD133,^77^ are ideal targets for potent immune therapy to reduce the GSC load. Recently, Wilms’ tumour 1 protein (WT1), a development-specific transcription factor that contributes to oncogenesis^116^ and used to be a TAA of other tumours,^117^^,^^118^ has been evaluated in several clinical trials on GBM patients (NCT03149003, NCT02649582, NCT01291420), suggesting that more GBM TAAs can be explored from existing TAAs of other tumours. However, one main limitation of TAA clinical usage is HLA restriction, which has over 25,000 variants in humans.^92^ Clinical trials for testing GBM TAAs are mainly restricted to the HLA-A2 allotype,^51^, ^52^, ^53^ and may lose function in patients with different HLA haplotypes. In addition to these existing TAAs, there is potential for multiple undefined TAA candidates worth exploring, such as alternative splicing or transposable elements.

**Table 1:** Current phase II/III clinical trials of GBM vaccines.

	Antigen Target	Antigen Platform	Antigen Classification	Phase	Status	NCT Identifier
Autologous GSC lysate	Autologous glioma stem-like cell antigens	DC	Personalized antigen	I/II	Completed	NCT00846456
	Autologous tumour and GSC lysate	DC	TSA + TAA	II/III	Unknown	NCT01759810
Autologous tumour lysate	Autologous tumour antigens	Uncertain	Personalized antigen	II	Completed	NCT00014573
	Autologous tumour lysate	Peptide	Personalized antigen	II/III	Not yet recruiting	NCT05685004
	Autologous tumour lysate	DC	Personalized antigen	I/II	Recruiting	NCT03879512
	Autologous tumour lysate	DC	Personalized antigen	I/II	Recruiting	NCT04801147
	Autologous tumour lysate	DC	Personalized antigen	II	Active	NCT01204684
	Autologous tumour lysate	DC	Personalized antigen	II	Active	NCT03400917
	Autologous tumour lysate	DC	Personalized antigen	II	Completed	NCT00323115
	Autologous tumour lysate	DC	Personalized antigen	II	Completed	NCT01213407
	Autologous tumour lysate	DC	Personalized antigen	II	Completed	NCT00576537
	Autologous tumour lysate	DC	Personalized antigen	II	Completed	NCT01006044
	Autologous tumour lysate	DC	Personalized antigen	II	Recruiting	NCT04523688
	Autologous tumour lysate	DC	Personalized antigen	II	Recruiting	NCT04115761
	Autologous tumour lysate	DC	Personalized antigen	II	Recruiting	NCT03395587
	Autologous tumour lysate	DC	Personalized antigen	II	Unknown	NCT02772094
	Autologous tumour lysate	DC	Personalized antigen	II	Withdrawn	NCT03014804
	Autologous tumour lysate	DC	Personalized antigen	III	Active	NCT00045968
	Autologous tumour lysate	DC	Personalized antigen	III	Not yet recruiting	NCT05100641
	Autologous tumour lysate	DC	Personalized antigen	III	Unknown	NCT04277221
EGFRvIII	EGFRvIII	Peptide	TSA	II	Completed	NCT00643097
	EGFRvIII	Peptide	TSA	II	Completed	NCT00458601
	EGFRvIII	Peptide	TSA	III	Completed	NCT01480479
	EGFRvIII, IL13Rα2, EphA2, Her2/neu, YKL-40	Peptide	TSA + TAA	II	Withdrawn	NCT02754362
GSC Antigen	GSC antigens	DC	TSA + TAA	II	Recruiting	NCT04888611
	GSC antigens	DC	TSA + TAA	II	Unknown	NCT01567202
	GSC antigens, survivin and hTERT	DC	TSA + TAA	II/III	Active	NCT03548571
Survivin	SurVaxM (survivin)	Peptide	TAA	II	Active	NCT02455557
	SurVaxM (survivin)	Peptide	TAA	II	Recruiting	NCT05163080
WT1	Wilms’ tumour protein 1 (WT1)	Peptide	TAA	III	Completed	NCT03149003
	Wilms’ tumour protein 1 (WT1)	DC	TAA	I/II	Completed	NCT01291420
	Wilms’ tumor protein 1 (WT1)	DC	TAA	I/II	Recruiting	NCT02649582
HSPPC-96	HSPPC-96	Peptide	TAA	I/II	Completed	NCT00293423
	HSPPC-96	Peptide	TAA	I/II	Recruiting	NCT03650257
	HSPPC-96	Peptide	TAA	II	Completed	NCT00905060
	HSPPC-96	Peptide	TAA	II	Completed	NCT03018288
	HSPPC-96	Peptide	TAA	II	Terminated	NCT01814813
Combined TAA	ICT-107 (MAGE-1, HER-2, AIM-2, TRP-2, gp100, IL13Rα2)	DC	TAA	II	Completed	NCT01280552
	IMA950 (BCAN, CSPG4, FABP7, IGF2BP3, NRCAM, NLGN4X, PTPRZ1, TNC, survivin, c-met)	Peptide	TAA	I/II	Active	NCT03665545
	IMA950 (BCAN, CSPG4, FABP7, IGF2BP3, NRCAM, NLGN4X, PTPRZ2, TNC, survivin, c-met)	Peptide	TAA	I/II	Completed	NCT01920191
	SL-701 (survivin, IL-13Rα2, and EphA2)	Peptide	TAA	I/II	Completed	NCT02078648
CMV	CMV (gB and pp65)	Virus vector	Pathogen-derived antigen	I/II	Recruiting	NCT03382977
	CMV (pp65)	DC	Pathogen-derived antigen	II	Active	NCT02465268
	CMV (pp65)	DC	Pathogen-derived antigen	II	Active	NCT03688178
	CMV (pp65)	DC	Pathogen-derived antigen	II	Completed	NCT02366728
	CMV (pp65)	DC	Pathogen-derived antigen	II	Terminated	NCT03927222
Other	Dendritic and Glioma Cells Fusion vaccine	DC	Personalized antigen	I/II	Recruiting	NCT04388033
	Telomerase (TERT)-derived helper peptides	Peptide	TAA	II	Active	NCT04280848
	VXM01 (VEGFR2)	Attenuated Salmonella bacteria	TAA	I/II	Active	NCT03750071
	EO2401 (IL13Rα2, BIRC5, FOXM1)	Peptide	TAA	I/II	Active	NCT04116658

#### TAAs from alternative splicing

Alternative splicing occurs in approximately 92%–95% of multi-exon genes in humans,^119^ whose changes reshape cancer-associated phenotypes by promoting angiogenesis,^120^ inducing cell proliferation,^121^ and avoiding apoptosis.^122^ Alternative splicing changes can be another tumorigenesis driver that is mutually exclusive of the mutation driver.^123^ Changes in the expression of core or auxiliary splicing factors in tumour cells generate oncogenic splicing variants, and a relatively small change in splicing factors or their downstream transcript abundance is sufficient to trigger oncogenic effects in cells.^124^ By aberrant alternative splicing events, tumour cells can generate abnormal proteins with gained/loss domains to remodel the intracellular protein–protein interaction (PPI) network, and these newly formed peptide sequences, especially located at the splicing junction, may be ideal candidates for alternative splicing-derived antigen vaccines.

#### TAAs from transposable elements

Epigenetic dysregulation in tumours lead to de-repression of transposable elements (TEs).^125^ As a result, the non-coding genome from TEs in normal adult somatic cells aberrantly translates into peptides in GBM cells.^126^ These tumour-associated peptides with potential immunogenicity can be further presented by MHC I molecules^127^ and may function as GBM TAA targets.

### Pathogen-derived antigen in GBM

#### Viral-derived antigen

Human cytomegalovirus (CMV) nucleic acids and proteins are initially distinctively detected in GBM tissues, but not in the surrounding normal brain, from >90% of patients.^128^^,^^129^ However, the presence of CMV in GBM cells is still under significant debate because some rigorously performed studies have failed to detect CMV proteins or DNAs in GBM clinical samples.^130^, ^131^, ^132^ Some of these failures may be due to sample preparation methodologies and detection strategies or fragmented/discontinuous viral genomes in the bulk short-read sequencing data generated from next-generation sequencing (NGS),^133^^,^^134^ which may be improved by optimizing the protocol^134^^,^^135^ or using long-read sequencing technology.^136^ Despite the debated CMV existence, some clinical trials have reported that targeting pp65 CMV immunodominant epitope with one injection of autologous tumour lysate-pulsed DC immediately invoked a CMV-specific CD8+ T cell response in GBM patients,^93^^,^^94^ revealing CMV viral antigen as an attractive exogenous immunotherapeutic target.^73^ Clinical trials conducted by the Khanna group in Australia reported that autologous CMV-specific T cell transduction is a safe adjuvant immunotherapy to improve the clinical outcomes of patients with primary^137^ and recurrent GBM.^138^ In addition, lysed CMV-infected tumour cells expose more tumour antigens to the TME,^138^ allowing APCs to present various antigens, thereby promoting antigen spreading.^39^ Despite the increasing expansion of primed CD8+ T cells, some issues related to the anti-CMV T cell strategy remain to be resolved before conducting further clinical trials, such as immune dysfunction and loss of susceptibility.^139^

#### Intratumoural bacteria

Intratumoural bacteria potentially induce strong immunogenic antitumor efficacies and have significantly extended survival rates with effective immunological memory in various murine cancer models.^140^^,^^141^ Bacteria-derived peptide-recognized memory T cells, which are pre-stimulated by bacteria-derived or tumour-derived antigens, exist in both peripheral blood and tumours.^142^ As a result of molecular mimicry,^143^ cross-reactivity between human tumour antigens and bacterial antigens allows tumour antigen recognition by CD4+ T cells^142^ and CD8+ cytotoxic T cells.^144^ These bacteria-derived peptides can be used in combination with other tumour vaccines. GBM contains distinct microbial communities compared with adjacent normal tissues.^145^ Both GBM tissues and cell lines present bacteria-specific peptides via MHC II molecules. After bacterial peptide stimulation, tumour-infiltrating lymphocytes secrete more pro-inflammatory cytokines and recruit CD8+ T cells to promote tumour-killing.^99^ For patients that hardly detect intratumoural bacterial nucleic acids or peptides, injecting tumour antigen-engineered commensal bacteria subcutaneously to elicit distant anti-tumour immune reactions is a novel and safe approach,^141^ and is worth exploring for GBM treatment. By lysing subcutaneously engineered bacteria, epidermal Langerhans cells further present tumour peptides in MHC molecules for T-cell priming, and then distant primed CD8+ T cells enter the circulation and infiltrate into tumours for cytotoxicity. This vaccination strategy avoids HLA restriction and tumour desertification,^146^ in which tumour cells disturb antigen uptake by DCs via various immunosuppressive mechanisms.

## Challenges for the GBM vaccine

**Fig. 2:**
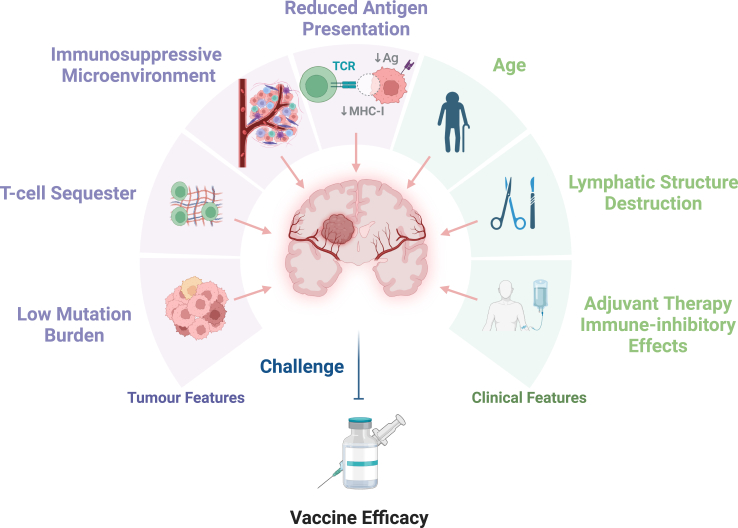
Challenges that hamper GBM vaccine efficacy. The purple means the challenges related to GBM tumour features. The green represents the challenges caused by the patient’s clinical features.

To identify additional GBM antigens, many studies have attempted to predict GBM neoantigens^147^^,^^148^ and TAAs^126^^,^^149^ from tumour lysates, peptides isolated from purified tumour MHC molecules,^150^ and next-generation sequencing analysis. Because existing GBM neoantigen predictions are mainly based on single nucleotide mutations, there are still plenty of GBM neoantigens derived from structural variants, frameshifts, and changes in chromosome structure waiting to be explored. Immunopeptidome applications help detect HLA-presented peptides on the tumour surface, accelerating GBM antigen prediction progression and increasing reliability.^113^^,^^150^ Apart from the lack of valid GBM antigens, other challenges need to be considered to improve the clinical outcome of vaccination (Fig. 2).

### Challenges from GBM tumour features

#### Low tumour mutation burden

CNS tumours, including GBM, which may initiate mainly from epigenetic lesions,^30^ have regularly low TMB.^28^^,^^29^ Because neoantigen presentation is a probabilistic process^29^ dependent on neoantigen abundance, few mutations can be present in GBM cells as neoepitopes^54^ for efficient autologous T-cell recognition. Although some neoantigens occasionally possess immunogenicity and are presented by APCs, only a minority of mutations are processed to MHC-presented neoepitopes that can be targeted by T cells.^151^, ^152^, ^153^ Among the presented neoantigens, most arise from passenger mutations and have limited potency because tumour subclones without these highly antigenic tumour-specific mutant peptides escape from T-cell-dependent immunoselection.^154^ Additionally, antigen spreading, a critical mechanism of immunotherapy response, occurs after therapeutic tumour vaccination.^39^ It is induced by increased tumour antigen exposure, which is attributed to the initial vaccine-mediated tumour lysis. A smaller antigen pool due to low TMB allows fewer immunogenic neoantigens to be exposed following vaccination, which may reduce the benefits from antigen spreading after immunotherapy.

#### T-cell sequester

In GBM patients, including treatment-naïve patients, it has been reported that the majority of T cells, including both CD4+ and CD8+ T cells, have been sequestered in the bone marrow due to tumour-imposed loss of S1P1,^155^ leading to insufficient T cell homing to secondary lymph organs or circulating in the peripheral blood, resulting in less T cell infiltration into GBM. Although high microvessel density correlates with glioma grade, these dysfunctional vessels are poorly perfused.^156^ Dysfunctional glioma vasculature hampers T cell recruitment and tertiary lymphoid structure (TLS) neogenesis,^157^^,^^158^ resulting in a hypoxic and immunosuppressive TME that aggravates with tumour grade. With increasing intratumoural hypoxia levels, more CD8+ cytotoxic T cells will be entrapped in pseudopalisading hypoxic zones formed by hypoxic GBM cells in immunocompetent hosts, limiting inflammatory spread.^159^ Sequestered CD8+ cytotoxic T cells will transform to immunosuppressive phenotype by experiencing hypoxia and interacting with trapped immunosuppressive tumour-associated macrophages (TAMs) that mainly migrate from peripheral blood. Hypoxia-associated T-cell sequesters may counteract the clinical benefits of bevacizumab therapy.^160^ Thus, before performing efficacy studies, when feasible, early-phase trials should evaluate antigen-targeted T-cell infiltration as it is an essential step in achieving a response. Poor infiltration of T-cells in many GBM remains a barrier to progress and future studies are warranted to understand and improve the infiltration and biodistribution of immune cells throughout GBM.

#### Immunosuppressive TME

In GBM, effector T cells gain exhaustion features that are attributed to various factors in the TME.^10^ IL-10 plays a crucial role in conferring immunosuppressive TME in GBM^161^ by inhibiting APCs, hampering T-cell proliferation, and inducing the activity of regulatory T (Treg) cells.^162^ It induces tolerogenic DC maturation, thereby promoting Treg differentiation.^70^ Previous studies have revealed that glioma patients exhibit an increased fraction of CD4+ Treg cells in both the blood^163^ and tumours.^164^ High levels of Treg cells decrease the T cell antitumor response by upregulating immune checkpoint molecules or secreting immunosuppressive cytokines,^54^^,^^165^ and treating mice with neutralizing antibodies against CD25 to eliminate the suppressive function of Tregs^166^ can rescue the inhibited cytotoxic T cell antitumor functions. Moreover, intratumoural Tregs have a distinct TCR repertoire that specifically recognizes tumour neoantigens^167^ or TAAs,^168^ suggesting that the tumour antigens may lead to tumour antigen-recognized Treg cell clonal expansion. Depleting Treg clones that recognize tumour antigens will promote anti-tumour responses of effector/memory T cells targeting identical tumour antigens.^168^ Tumour-associated myeloid cells (TAMC), consisting of tumour-associated macrophages (TAM) and myeloid-derived suppressor cells (MDSC), are another critical component in the formation of an immunosuppressive TME in GBM. TAMCs account for 30%–50% of the mass in GBM^169^^,^^170^ and are the major source of PD-L1 expression in GBM TME to hamper adaptive immune response.^171^ Targeting PD-L1 on GBM TAMCs can profoundly reverse their immune inhibitory effects, reactivate CD8+ T cells, and prolong OS in GBM mouse models.^172^ Approximately 85% of TAMs originate from peripheral blood (bone marrow-derived macrophage/monocyte, BMDM) and are recruited to perivascular areas in GBM with the remaining resident TAMs (microglia) located surrounding the GBM tumour mass.^173^ TAMs from BMDMs (macrophages) preferentially exhibit nonpolarized (undifferentiated) or immunosuppressive phenotypes,^174^ and their abundance in the GBM TME may be associated with ICB resistance and poor T-cell infiltration.^175^ Resident TAMs (microglia) can promote tumour immune evasion by mTOR signalling to hinder effector T cell infiltration and proliferation within the GBM TME.^174^ Co-culture of human CD14+ monocytes from healthy donors with GBM cells induces MDSC-like properties in these monocytes.^176^ A previous study depicted MDSC accumulation in the peripheral blood of GBM patients.^176^^,^^177^ As a result of the upregulated GM-CSF expression in the GBM TME, GBM-infiltrated myeloid cells increase their Interleukin-4 receptor-α (IL-4Rα) expression, which mediates IL-13-induced production of arginase, thereby suppressing T-cell proliferation and function.^178^ The inhibition of T-cell function can be significantly restored after removing MDSCs from peripheral blood.^177^ Additionally, for patients who accept tumour vaccination, T cells persistently accumulate at the vaccination site owing to the antigen concentration gradient. Constant interaction with the vaccine for at least five days drives T cell anergy mediated by IFN-γ and MDSCs.^179^ Although short-term IFN-γ secretion can promote MHC I complex expression in GBM cells,^55^ accumulation of IFN-γ induces T cell anergy and upregulates the expression of various immune checkpoint genes,^179^ promoting a profoundly exhausted phenotype of T cells during long-term vaccination courses.^55^

#### Antigen loss and reduced antigen processing and presentation

In addition to the overexpression of checkpoint inhibitor molecules,^180^ antigen loss, resulting from the downregulation of antigen-originated proteins or antigen presentation,^181^ and degradation of MHC molecules,^81^ are other key challenges for successful immunotherapy. GBM is one of the refractory tumours with high heterogeneity,^182^^,^^183^ especially at the molecular level.^112^^,^^184^ Apart from diverse bulk^184^^,^^185^ and single-cell^112^^,^^186^ transcriptional subtypes, the tumour exhibits high plasticity that enables its dynamic phenotype to change to adjust to variable pressures,^29^^,^^187^ leading to most GBM antigens being highly changeable private antigens, even in different regions of the same tumour.^188^ The currently evaluated GBM neoantigen vaccine targeting EGFRvIII face the risk of antigen loss, and the phase III clinical trial of rindopepimut reported that approximately 60% of GBM patients lost EGFRvIII expression during recurrence.^47^ The disappearance of clonal or subclonal mutations during GBM clonal evolution^98^ mediated by chromosomal deletions and loss of heterozygosity (LOH),^189^ abnormal chromosomal structure,^190^^,^^191^ and epigenetic modifications contribute to GBM antigen loss, thereby decreasing its immunogenic antigenic protein expression. Furthermore, during post-surgery recurrence, the immunoediting effect^99^ and the balance between immune supervision function and tumorigenesis consistently sculpts the tumour antigen pool. During the escape phase, the loss of abundant immunogenic antigens^192^ and the dominance of immunosuppressive features challenge immunotherapy efficacy. Of note, the kinetics of antigenic change over time with therapy pose challenges in vaccine design. One group is now trying to solve this problem by using immunogenic cell death-induced serum-derived antigenic extracellular microvesicles and apoptotic bodies, which results in an actualized antigenic profile at the time of effective vaccination.^15^ Mutation, downregulation, or release of MHC molecules is another immune evasion mechanism in GBM.^150^^,^^193^^,^^194^ Loss-of-function mutations or LOH^195^ of MHC-I and beta-2-microglobulin (B2M) are frequently enriched across tumours^181^^,^^196^ and the adaptive immune system promotes evolutionary selection by killing tumour cells that present immunogenic antigens. During the proposed equilibrium and escape phase of the GBM “immunoediting” process,^99^ the tumour cells that lost immunogenic antigen presentation survived under adaptive immune system supervision. As evidence for the importance of MHC expression, upregulating glioma-derived exosomal MHC-I expression can assist CD8+ T cells in recognizing tumour cells, interrupting glioma cells from evading immunosurveillance.^197^ Cooperatively, a large scale of stochastic mutation induced by TMZ^29^^,^^98^ will accelerate this selection progression to promote surface immunogenic MHC-antigen complex loss. Thus, clonal evolution,^98^ subtype switching,^198^ and LOH in MHC and B2M genes^199^ after treatment potentially alter the GBM antigen repertoire longitudinally, resulting in antigen loss for vaccine therapy.^47^

### Challenges from GBM patient clinical features

#### Age

GBM frequently occurs in the older population (median age:64 years), with the highest occurrence between 75 and 84 years,^200^^,^^201^ and patients at younger ages receive more benefits from tumour vaccination.^50^ Natural changes in the immune system with age can negatively affect the efficacy of tumour vaccination. Memory T cells, especially virtual memory T cells (T_vm_),^202^ which have a memory phenotype in the absence of foreign antigens, accumulate with age, erode the naïve CD8+ T cell compartment, and restrict the T-cell receptor (TCR) pool and neoplastic antigen recognition in older patients. This naïve:memory ratio imbalance of T cells has been attributed to thymus involution.^203^ During age-related thymus involution, thymocytes decrease and are replaced by adipocytes, resulting in diminished peripheral T-cell repertoire diversity and immunosenescence,^204^ compromising the detection and response to new stimulants. Deep cervical lymph nodes (DcLNs), considered the main secondary lymph organ response to CNS tumours, also experience age-related atrophy,^205^ thereby further shrinking the T cell compartment. Dural lymph vessel cerebrospinal fluid (CSF) drainage function decreases with age. Old age is associated with dysmorphology, increased lymphatic channel thickness,^205^ and dysfunction of dural lymph vessels.^206^ These alterations impair metabolic waste clearance from the CNS,^207^ and hamper the circulation of GBM antigens through the lymph as well.^208^ Simultaneously, age-related inflammageing condition^209^ may further inhibit antigen-specific immunity and reduce the vaccination effectiveness.^210^ In a phase I/II trial of oncolytic DNX-2401 virotherapy plus ICB in recurrent GBM, patients with complete response (CR) were under 30 years old suggesting age as a potential beneficial factor.^34^ Consequently, measures to enhance the basal immune reactivity of elderly GBM patients may be needed to render tumour vaccine therapy more efficacious. The Thymus Regeneration, Immunorestoration, and Insulin Mitigation (TRIIM) clinical trial results^211^ point to a future direction to overcome age-associated unfavourable factors for enhancing anti-tumour immunity. It succeeded in causing thymic regeneration and an expansion of the pool of naive T cells by a combination of growth hormone, metformin, and dehydroepiandrosterone in nine healthy 51–65-year-old men for one year. This may be a potential method for enhancing the immune response following vaccination in GBM patients.

#### Postsurgical lymphatic structure destruction

The brain was considered devoid of lymphatics until the exact dural lymphatic vessels were observed in 2015.^212^ The discovery of structural lymphatic vessels^206^^,^^212^ and the glymphatic system^213^ in the brain has helped researchers understand the accurate process of tumour antigen presentation from parenchymal to dcLNs.^205^^,^^212^^,^^214^ Surgery is the primary treatment option for GBM patients. For visual exposure, GBM resection involves incision and peeling of the membranes above the tumour, perturbing the proximal structures around the tumour. The procedure may irreversibly destroy the dural lymph vessel^206^^,^^212^ and the subarachnoid lymphatic-like membrane (SLYM)^215^ proximal to the primary site, where APCs, particularly DCs, localize or migrate for immune surveillance. Due to lymph structure damage, tumour antigens from the post-resection remaining tumour that invade the surrounding brain parenchyma may not adequately reach the secondary lymph nodes. This is supported by data demonstrating that CNS lymphatic drainage and neuroinflammation are regulated by meningeal lymphatic vasculature.^214^ Meanwhile, the postoperative inflammageing condition of patients may exacerbate immunosuppression.^209^^,^^210^ More work is warranted to identify the precise mechanism of brain tumour antigen drainage into the lymph nodes.

#### Adjuvant therapies decrease T cell clone diversity and function

Apart from age-induced immunosenescence, multiple antineoplastic therapies, such as radiotherapy and chemotherapy, deplete lymphoid precursors, induce thymocyte apoptosis,^216^ and accelerate thymus involution,^217^ thereby damaging the normal T cell development process and leading to prolonged deficiency in the adaptive immune system.^218^ Antigen-induced clonal expansion in lymph nodes cannot resolve this impairment.^219^ TMZ is the primary alkylating agent used for GBM adjuvant chemotherapy. Despite significant survival-prolonged benefits,^1^^,^^220^ TMZ treatment promotes GBM gain by more than 10 times mutations stochastically at recurrence (mainly C>T transitions),^29^^,^^98^ including the DNA mismatch repair (MMR)-encoding genes (MSH2, MSH5, MSH6, MHS4, MLH1, MLH2, PMS1, and PMS2),^221^ whose deficiency contributes to post-treatment hypermutation^222^ and TMZ resistance.^223^ By increasing genome instability within GBM, TMZ treatment limits the effectiveness of antigen-targeting therapies.^224^ Genes containing TMZ-induced hypermutated loci are more highly expressed in recurrent GBM, but the increased neoantigen load fails to improve the effects of immunotherapy.^222^ Additionally, standard TMZ eradicates both proliferating CD4+ and CD8+ T cells and promotes CD8+ T cell exhaustion, thereby hindering immunotherapy effectiveness.^224^ Edema is a common complication of GBM. To limit the progression of peritumoral edema, physicians often choose corticosteroids. Theoretically, corticosteroid mediated depletion of immune cells may counteract the anti-tumour effects of tumour vaccines,^55^^,^^225^ however, there is no phase III clinical trial data yet definitely reporting the adverse effect on GBM patients’ survival. Alternatives to steroids should also be considered in conjunction with GBM vaccines. Bevacizumab performs well in controlling brain edema.^226^ Combining GBM vaccine therapy with bevacizumab is a feasible strategy to control edema as a steroid alternative.^227^

## Perspective: future direction and opportunities to improve GBM tumour vaccine efficacy

**Fig. 3:**
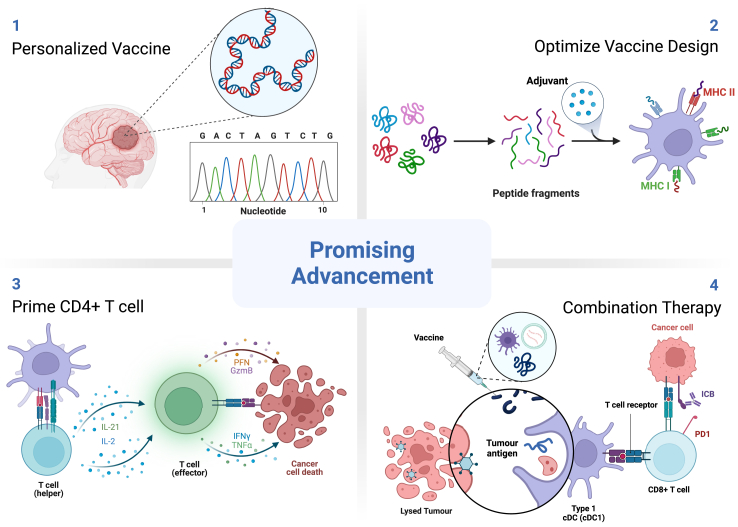
Promising advancements for GBM vaccine efficacy.

GBM vaccine is a feasible approach to improve patients’ clinical outcomes with SOC. Although the previously mentioned challenges can hamper the efficacy of current GBM vaccines, there are many promising opportunities to improve their therapeutic effects (Fig. 3).

### Personalized multi-peptide vaccine

Due to interpatient heterogeneity, except for some common driver mutations, such as EGFRvIII mutation, among GBM patients vary from each other, resulting in the majority of neoantigens derived from these mutations being seldom shared. Testing of personalized vaccines for GBM patients began over 20 years ago, which harvested tumour lysates, primed T cells from the peripheral blood of GBM patient, stimulated and expanded them in vitro, and then infused tumour antigen-target T cells back into the patient.^228^ This clinical trial reported an increased antitumor immune response but no survival benefit in the test cohort (median OS:12 months). Compared to priming T cells with all tumour lysate proteins unselectively, personalized neoantigen design from bioinformatic prediction shows potential for improving GBM patient clinical vaccine efficacy.^55^ Meanwhile, by integrating diverse private tumour antigens, a large proportion of tumour cells that present different antigens will be recognized by primed T cells, thereby effectively reducing the tumour load, and counteracting the antigen loss risk. The multi-peptide combination strategy has been evaluated to improve the prognosis of patients with newly diagnosed GBM.^54^^,^^55^ Generally, neoantigens are expected to be highly immunogenic, but tend to be private (most mutations are unique to individual tumours; exceptions include some well-known driver mutations), whereas TAAs are widely applicable with less immunogenicity.^108^ Combining both neoantigens and TAAs in GBM patient vaccination can boost the vaccine effect compared with the monotype vaccine. Personalized multi-peptide vaccine design containing both neoantigens and TAAs detected by exome sequencing, transcriptome sequencing, and HLA immunopeptidomes may yield a favourable CD8 cytotoxic T cell intratumoural infiltration and survival extension.^54^ In two small-scale clinical trials of the GBM personalized vaccine, two study groups tested the efficacy of personalized antigens with/without TAAs in aged cohorts (median age:52.5, 65 years) of newly diagnosed GBM.^54^^,^^55^ Even with more mutations, the cohort receiving a personalized vaccine composed of neoantigens showed shorter OS than the other cohort being vaccinated by both neoantigens and TAAs (median OS:29 months vs. 16.8 months), indicating TAA supplementation in the personalized multi-peptide design can further extend patients’ survival. Additionally, a therapeutic vaccine prepared via GSC mRNA-transfected DC can significantly prolong PFS in GBM patients,^229^ which offers an additional option for personalized multi-peptide vaccine design.

### Optimize vaccine peptide design

*Long-length peptide designs* can induce minimal T-cell sequestration at the vaccination site, attenuate T-cell exhaustion, and enhance anti-tumour responses after tumour vaccination.^179^ On the one hand, long peptides require cleavage in vivo by APCs, preventing overwhelming peptide presentation by various cells, including B cells, thus trapping fewer T cells at the vaccination site. On the other hand, it allows injected peptides to be trimmed into various short-length peptides to fit the binding grooves of multiple MHC allotypes, including MHC I (through cross-presentation) and MHC II molecules.^49^ In addition to direct tumour cell killing mediated by MHC-I-peptide primed CD8+ T cells, MHC–II–peptide primed CD4+ T cells can provide sufficient co-stimulatory signals to CD8+ T cells to enhance their cytotoxicity.^230^^,^^231^

*Short-lived vaccine*’*s* fast degradation of the vaccine peptides, attributed to the lack of persistent peptide-holding adjuvants, decreases the probability of T-cell sequestration at the vaccination site and T-cell apoptosis mediated by vaccine-induced high levels of IFN-γ.^179^ Continued tumour antigen exposure impairs T-cell effector function and drives T-cell epigenetic remodelling to a dysfunctional state.^232^ Short-lived vaccines can promote T cell localization to tumours, prevent T cell dysfunction, and attenuate T cell apoptosis at the expense of reduced T cell priming efficiency.^179^ However, this shortcoming can be rescued by combination with immunostimulatory supplements.

### Vaccine adjuvant and neoadjuvant

Vaccine adjuvants refer to immunostimulatory components, given in addition to the antigen, to enhance the durability and magnitude of the immune response. Owing to the low immunogenicity of cancer peptide vaccines, combination with adjuvants is likely to improve the induction of effective antigen-targeting immune responses.^233^^,^^234^ After in vivo injection with tumour antigens, the adjuvant typically activates the innate immune system by introducing pathogen-associated molecular patterns (PAMPs) or damage-associated molecular patterns (DAMPs), inducing cytokine production, and recruiting innate immune cells to enhance adaptive immune responses.^235^ Insoluble aluminium salt (alum) is the most commonly used adjuvant in humans and in infectious disease vaccines such as hepatitis B^236^ and malaria.^237^ Alum mainly promotes the Th2-type response,^235^ making it less suitable as a tumour vaccine complement, in which a proinflammatory response is the typical goal. For tumour vaccines, incomplete Freund’s adjuvant (IFA) is widely used in clinical research on various cancers, which can protect against antigen degradation and release antigens slowly to APCs.^238^, ^239^, ^240^ However, it can cause T-cell sequestration at the vaccination site and destroy long-lived CD8+ T-cell immunity.^179^ Adjuvants on the market used in prophylactic vaccines are typically thought to not effectively induce a CD8+ T-cell response.^235^ AS04, an aluminium salt absorbing 3-O-desacyl-4′-monophosphoryl lipid A (MPL), which is a detoxified form of lipopolysaccharide (LPS) extracted from *Salmonella Minnesota*, can provoke a prominent Th1-type response, suggesting the added value of the Toll-like receptor (TLR) agonist MPL (TLR4 agonist) as a human cancer vaccine adjuvant.^241^ Targeting TLRs has great potential as tumour vaccine adjuvants. In mice, delivering antigens with TLRs ligands can induce antigen-specific CD8+ T-cell responses.^242^^,^^243^ A phase I clinical trial in GBM patients reported significantly increased T cell chemoattraction after complementing CpG-oligodeoxynucleotides (CpG-ODN), a TLR9 ligand, with a peptide vaccine from hTERT.^244^ Polyinosinic–polycytidylic acid (poly (I:C)), a TLR3 agonist, potently induces antitumor CD8+ T cytotoxic responses.^245^ Stabilizing this synthetic double-stranded RNA (poly (I:C)) with poly-L-lysine forms its RNase-resistant analog, ploy-ICLC, which can be a reliable and authentic microbial mimic for upregulating interferon and inflammasome signals in innate immune cells.^246^ Poly-ICLC is often used in GBM vaccine clinical trials (NCT03665545, NCT05557240, NCT02078648, NCT02510950, NCT01920191) with or without granulocyte-macrophage colony-stimulating factor (GM-CSF) (NCT02149225), a widely used adjuvant,^247^ to advance APC function and boost antitumor response.^248^^,^^249^ The stimulator of interferon genes (STING) pathway is a sensor of central cellular cytosolic double-stranded DNA (dsDNA), which can be activated by intrinsic self-dsDNA release from cancer cells. Its activation attenuates the early progression of tumour cells by mediating immune cell infiltration, such as T cells and natural killer (NK) cells, and upregulating proinflammatory cytokines, such as type I IFNs and chemokine secretion.^250^ STING agonists are another potential adjuvant for tumour vaccine activation.^250^ Its usage significantly enhances T-cell infiltration and inhibits tumour size in a dose-dependent manner compared with GM-CSF alone.^251^ In addition to the available adjuvants mentioned above, novel adjuvants for tissue damage, cell death, metabolic modulation, and epigenetic modulation are worth further exploration.^235^

### Prime CD4 T cells

The CD4+ helper T cells, are important for tumour antigen specific CD8+ T cell population maintenance. After presenting tumour antigens, DCs prime CD4+ T cells, resulting in IL-2 production, which facilitates T cell proliferation and expansion.^70^ CD8+ T cells primed by interacting with both DCs and activated CD4+ T cells will gain a robust primary induction, including long-term maintenance and higher proliferative ability when suffering a secondary challenge, compared to those primed without CD4+ T cells.^252^ Primed CD4+ T cells promote CD8+ T cells to gain a cytotoxic phenotype by IL-21 secretion against differentiation to exhaustion subclones.^253^^,^^254^ A recent study identified a cellular triad associated with antitumor efficacy.^255^ The intratumoural cellular triad consists of DCs, CD4+ T cells, and CD8+ T cells. The triads of spatially attracted CD4+ T cells and DCs to the periphery of CD8+ T cells promote local CD8+ T cell differentiation into effector T cells and enhance CD8+ T cell cytotoxic potency. Antigen-induced antitumor responses require both tumour-antigen-primed CD4+ and CD8+ T cells.^230^ CD4+ T cell priming guarantees effective tumour rejection during vaccination.^254^^,^^256^ Disregarding CD4+ T cells, the level of CD8+ T cell infiltration alone is not a significant prognostic factor in glioma patients,^257^ revealing the importance of CD4+ T cells which has been underestimated in previous glioma antigen studies. GBM vaccine expanded both CD4+ and CD8+ T cells. Traditionally, TAAs have been designed to elicit sustained responses of effector and central memory CD8+ T cells. A recent study targeting a neoantigen, IDH1 R132H,^49^^,^^258^ was developed to induce CD4+ T cells of T helper Type 1.^54^ Thus, the CD4+ T cell response is vital to neoantigen-induced antitumor immunity, whose expanded TCR clone can react to IDH R132H.^49^ In addition to supporting CD8+ T cell cytotoxicity as a helper or regulator, the primary antitumor immunity response of CD4+ T cells is also non-negligible.^259^^,^^260^ CD4+ T cells effectively eliminated MHC-deficient tumour cells. For example, CD8+ T cells are unable to kill MHC-I-deficient tumour cells, whereas CD4+ T cells can eradicate MHC-I-downregulated tumour cells in cooperation with the innate immune system,^259^^,^^261^ or mediate CD8+ T cell anti-tumour response through CD40L signalling to kill tumour cells that lack MHC II molecule expression.^256^^,^^262^ Additionally, CD4+ T cells can elicit cytotoxicity to kill tumour cells in an MHC II-dependence manner.^263^, ^264^, ^265^ After priming, cytotoxic CD4+ T cell clones expand and enrich within tumours with various inflammatory cytokine secretions, such as IFN-γ and TNF-α, functioning as cytotoxic T cells. Consequently, considering MHC I and MHC II molecules’ compatible peptides and their TCR avidity together when designing a GBM vaccine can improve future vaccination efficacy.

### Combination therapy

Long-term vaccine usage upregulates immune checkpoint gene expression owing to the accumulation of IFN-γ. To combat this immunosuppressive mechanism, ICB is an ideal complement to vaccines. Combined ICB therapy augments vaccine anti-tumour efficacy in both a preclinical murine cancer model^266^ and a clinical trial,^267^ and was even able to induce a rapid and durable complete response in tumour patients with metastasis.^268^ ICB combination with neoantigen vaccine augments survival benefits in an ICB-resistant GBM murine model.^269^ A small-scale clinical trial on recurrent GBM (n = 3 patients/group) revealed that neoadjuvant ICB plus DC vaccine treatment prolonged the patient OS compared with treatment with DC vaccine without neoadjuvant ICB (median OS:15.3 vs 8 months) (NCT02529072). Another study reported on a GBM case with long-term combinational therapy consisting of autologous DC vaccines expressing mRNAs encoding both TAAs and neoantigens, with anti-PD-1 and poly I:C in which the patient exhibited 69 months of progression-free survival.^270^ Other clinical trials of ICB and vaccine combinations for GBM treatment are ongoing (NCT02287428, NCT04888611, NCT04201873, NCT03018288), and no results have been reported. Another issue for immunotherapy in GBM is the ‘cold’-immune excluded TME.^271^ Intratumoural injection of oncolytic virus, DNX-2401, can transform the ‘cold’ GBM TME to ‘hot’ TME in patients with recurrent GBM by lysing tumour cells and exposing tumour antigens,^272^ benefiting GBM regression even to a best response of CR in two patients when combined with intravenous ICB.^34^ A phase I/II clinical trial reported favourable survival extension and tumour regression after oncolytic virus treatment with expected blood–brain barrier (BBB) penetration in an intravenous delivery cohort.^273^ Despite the direct virus-mediated pro-antitumor effect, arming viruses with immunoregulatory genes can confer additional antitumor functions. The Yu group inserted an extra sequence that encodes anti-CD47 IgG1 or IgG4 antibodies into the oncolytic virus, resulting in anti-CD47 antibody secretion from infected GBM cells, which blocked the phagocytosis inhibitory immune checkpoint.^274^ In addition to directly lysing tumour cells, the oncolytic virus activates innate immune cells by translating anti-CD47 antibodies, thereby improving the antitumor response and survival. Other studies evaluating armed oncolytic viruses for GBM are ongoing.^275^ This technique offers the possibility of integrating tumour antigens with oncolytic viruses to enhance their antitumor properties. Additionally, a multiphase combined treatment approach may be a promising regimen complement after SOC.^276^ During chemotherapy and oncolytic virus treatment, the TME can transform from ‘cold’ to ‘hot’, with tumour-lysed antigens exposed in the TME. These tumour-lysed antigens are important sources of ICD-induced antigens. The efficacy of subsequent DC vaccine therapy will be boosted by diverse ICD-induced antigens in addition to its preload antigens.^15^^,^^276^

## Conclusion

GBM tumour vaccination holds promise as a complementary therapy to SOC. Compared to single-peptide vaccination, personalized multi-peptide vaccines consisting of neoantigens and TAAs exhibit better tumour regression and stronger anti-tumour responses. Due to the unique epigenetic mechanism of gliomagenesis, neoantigens are likely to be limited in number for GBM immunotherapy, and TAAs and pathogen-derived antigens may be useful in increasing the arsenal of vaccine antigens. Patient age, immune fitness, and treatment regimens can affect the efficacy of GBM vaccines. The optimal combination of tumour vaccines and SOC will be the focus of future clinical applications of GBM tumour vaccines. Optimizing vaccine design (antigen and adjuvant selection), concentrating on the role of CD4+ T cells in tumour vaccines, and combination immunotherapy will become new keys to increasing the efficacy of GBM vaccines.

### Outstanding questions

Tumour vaccine is a potent strategy to improve the therapeutic effect of patients with GBM after SOC. As a malignant tumour possibly derived from an abnormal epigenome, GBM has low TMB and few neoantigens, which may hamper the efficacy of immunotherapy. Various clinical trials on GBM vaccines reported unsatisfactory results for few clinical benefits. Optimizing vaccine design, including personalized antigen selection, multi-antigen targeting, and improving vaccine platform or adjuvant, will be future topics for improving GBM vaccine efficacy. Another challenge is activating robust anti-tumour response in patients against the immunosuppressive effects of GBM. How to strengthen a GBM patient’s immunity weakened by age, treatment, or tumour immune escape mechanism is worth considering when performing tumour vaccine therapy. Tumour vaccine works well when combining to other immunotherapies, such as ICB. Combinational treatment and immunizing premalignant LGG patients are novel aspects of improving vaccine treatment regime design. While there are numerous established and presumed advantages and disadvantages of each vaccine platform (mRNA vs. peptide, vs DC loaded, vs viral vector) and antigen source, ongoing and future trials should empirically determine the difference between each.

**Search strategy and selection criteria**

Data for this Review were identified by searches of MEDLINE, PubMed, and references from relevant articles using the search terms “glioblastoma”, “vaccine”, “antigen”, “clinical trial”, and “immunotherapy”. Abstracts and reports from meetings were included only when they were related directly to previously published work. Only articles published in English between 1996 and 2023 were included.

## Contributors

Z. X: Conceptualization, data curation, investigation, visualization, writing - original draft, writing - review & editing. I. R: Conceptualization, investigation, writing - review & editing. M. O: Conceptualization, writing - review & editing. H. O: Conceptualization, writing - review & editing. X. L: supervision, Conceptualization, writing - review & editing. G. K: Conceptualization, funding acquisition, project administration, resources, supervision, writing - review & editing. All authors read and approved the final version of the manuscript.

## Declaration of interests

All authors have nothing to disclosure.
